# Interleukin-8 (IL-8) levels in gingival crevicular fluid during root canal treatment of molar teeth with symptomatic irreversible pulpitis: impact of varying sodium hypochlorite concentrations

**DOI:** 10.1186/s12903-024-04128-6

**Published:** 2024-03-27

**Authors:** Esin Özlek, Gizem Kadı, Nasser Shoshaa, Yusuf Saed, İsmet Meydan

**Affiliations:** 1 Department of Endodontology, Faculty of Dentistry, Van Yuzuncu Yil University, Van, Turkey; 2 Department of Endodontology, Faculty of Dentistry, Altinbas University, İstanbul, Turkey; 3 Department of Endodontology, Faculty of Dentistry, Arab American University, Jenin, Palestine; 4 Department of Chemistry, Faculty of Science, Van Yuzuncu Yil University, Van, Turkey

**Keywords:** Interleukin-8, Gingival cervicular fluid, Sodium hypochlorite

## Abstract

**Background:**

The aim of this study was to evaluate the effect of the use of different NaOCl concentrations (1%, 2.5%, and 5.25%) during root canal treatment of molar teeth with symptomatic irreversible pulpitis on the change of the IL-8 level in gingival crevicular fluid (GFC).

**Methods:**

GCF sampling was performed on experimental tooth with irreversible pulpitis before and after treatment and also contralateral healthy tooth of 54 patients. The patients were divided into three groups according to concentration of NaOCl solution (*n* = 18); 1%, 2.5%, and 5.25% NaOCl solution. GCF sampling from experimental teeth was repeated one week after root canal treatment. Statistical analysis was performed using Mann-Whitney U, Wilcoxon test, one-way ANOVA and Pearson correlation analysis.

**Results:**

There was a significant correlation between IL-8 levels in GCF samples taken from teeth with pulpitis before treatment and from healthy contralateral teeth (*p* = .000). Furthermore, the pretreatment IL-8 level was significantly higher than the posttreatment IL-8 level(*p* < .05). The effect of the NaOCl concentration on the change in IL-8 level betweeen pre and post treatment was not found statistically significant (*p* > .05).

**Conclusions:**

The use of NaOCl during root canal treatment can effectively reduce the levels of IL-8 in GCF and improve clinical outcomes.

**Trial registration:**

This study was registred in the Institutional Review Board and the Ethics Committee of the University (No:11) on 15/12/2021.

## Introduction

Cytokines are low molecular weight glycoproteins that play a role in important biological events such as cellular growth, inflammation, immunity, tissue repair, and hematopoiesis. They are released by immune cells (lymphocytes, macrophages, granulocytes, and neutrophils), particularly by mast cells (endothelial, epithelial cells and fibroblasts) [1, 2]. Cytokines have a balancing function between the immune and inflammatory responses and are divided into two groups as inflammatory and antiinflammatory cytokines. The proinflammatory cytokine group includes IL-2, IL-6, IL-8, IFN-γ, and TNF-α, and the antiinflammatory group includes IL-4, IL-10, and IL-13 [3–5].

IL-8 plays an important role in the pathogenesis of inflammation by managing neutrophil activation and chemotaxis. IL-8 is resistant to heat and proteolysis and thus maintains its function in sites of inflammation [6, 7]. Studies have shown that the IL-8 level in the gingival crevicular fluid (GCF) dramatically increases in teeth with symptomatic pulpitis compared to healthy teeth and is a potential marker for irreversible pulpitis [6, 8, 9]. Thanks to its superior antimicrobial activity and organic tissue solvent ability, sodium hypochloride (NaOCI) has been used in endodontic treatments for years. Despite all its advantages, however, it has some destructive effects against the chemical components and mechanical properties of dentin and cytotoxic effects on periradicular tissues. It is commonly used in concentrations of 0.5–5.25%, and studies have reported that its detrimental effects increase at higher concentrations [10]. Although there are many studies assessing the efficacy of different concentrations of NaOCl, a consensus is yet to be reached for its optimum concentration [11]. Most studies evaluating the efficacy of NaOCl concentration have been conducted in vitro, and no study has assessed its effects on the level of cytokines, the markers of inflammation. The aim of this study was to evaluate the effect of the use of different NaOCl concentrations (1%, 2.5%, and 5.25%) during root canal treatment (RCT) of teeth with symptomatic irreversible pulpitis on the change of the IL-8 level. The null hypothesis of the study was that NaOCl solution at different concentrations has no effect on the change of IL-8 level.

## Methodology

### Study design and setting

This randomised clinical trial was written according to Preferred Reporting Items for Randomised Trials in Endodontics (PRIRATE) 2020 guidelines [12]. Ethical approval for the study was obtained from the Institutional Review Board and the Ethics Committee of the University (11–15/12/2021). The Consolidated Standards of Reporting Trials (CONSORT) guidelines were adhered to in this clinical trial, and the study protocol was registered on www.clinicaltrial.gov (Identifier: NCT05277246) and first posted on 14/03/2022.

Sample size was calculated using Power and Sample Size Calculation software version 3.1.2 based on an error of α = 0.05, a power of 0.85, and effect size of 0.5 [13]. A minimum of 16 patients in each group, making a total of 48 patients, were required. This study included volunteering patients, and all patients signed an informed consent form after being informed about the objectives, procedures, benefits, and potential risks of the study.

### Patient selection

Patients who were admitted to the Department of Endodontics with pain originating from mandibular molar teeth and diagnosed with symptomatic irreversible pulpitis, and who needed canal treatment were considered potential candidates for this study. The study included patients who were older than 18 years, free of any systemic disease, and had not taken any antibiotic or analgesic within 10 days prior to the treatment. All teeth included in the study gave a prolonged response to the thermal test (EndoIce; Coltene/Whaledent Inc, Altstätten, Switzerland) and electric pulp test (Analytic Technology Corp. Redmond, WA, USA). In addition, when the pulp chamber was entered during the RCT, abundant bleeding from the pulp was observed. Patients with gingivitis who had gingival bleeding, or those who had gingival recession exceeding 3 mm, diffuse periodontitis with a deep pocket and tooth mobility, absent contralateral tooth, and teeth with periapical lesion were excluded. The pain intensity of each patient was recorded on a 10-cm visual analog scale (VAS) with “no pain” and “severe pain” scores [3].

### Randomization and blinding

The patients were randomly assigned to one of three groups using an online program (www.randomizer.org). Patients were blinded of their assigned group, but due to the nature of the study, the clinian was not blinded.

### Sample collection

To obtain GCF samples, the tooth to be treated and the contralateral toothe were isolated using a cotton roll and a saliva absorber. Supragingival plaque was carefully removed with a periodontal curette, and the teeth were dried with air-water spray for 10 s. A periopaper strip (Oraflow, Smithtown, NY, USA) was gently inserted into the mesial interproxal gingival groove of the relevant tooth until resistance was felt, and remained in place for 1 min. Any samples contaminated with blood or saliva were excluded. The periopaper strips were placed in 1.5 mL Eppendorf tubes (Labosel, Istanbul, Turkey) and stored at -80 ^o^C until further analysis [3].

The patients underwent alveolaris inferior nerve block and buccal infiltration anesthesia using 2 ml of articaine hydrochloride local anesthetic containing 1:100.000 adrenaline (Ultraver DS fort; Haver, İstanbul, Turkey). Rubber dam isolation was carried out on the relevant teeth and the endodontic access cavity was prepared using a sterile diamond roundel drill. The working length (WL) was determined using an apex locator (Propex Pixi, Dentsply Maillefer) and confirmed to be 0.5-1 mm shorter than the radiographic apex by periapical radiographs.

Root canals were shaped with the crown down technique, with an X-Smart Plus (Dentsply Maillefer, Ballaigues, Switzerland) endodontic motor and EndoArt (İnci Dental, İstanbul, Turkey) nickel titanium rotary file system. In accordance with the manufacturer’s recommendations, the root canals were shaped using an SX file with 300 rpm and a torque of 3.0 Ncm and shaping files with 300 rpm and a torque of 1.5 Ncm (#15(0.4), #20(0.4)). The mesial canals were then shaped using #25(0.6) finishing files with 300 rpm and a torque of 2.5 Ncm. The shaping process of the distal canals was completed using #30(0.6) finishing files with the same velocity and torque settings as the mesial canals. To prevent torsional overload when resistance was encountered during shaping, the files were withdrawn from the canal, and the apical opening was probed with a #10 K type file. The canals were irrigated, and the procedure was resumed. These steps were repeated until working lenght was reached. The patients were randomly assigned to one of three groups based on the concentration of the NaOCl solution used during the shaping process:

#### Group 1 (5.25% NaOCl)

Irrigation with 2 ml 5.25% NaOCl (Microvem AF, İstanbul, Turkey) solution was performed for 1 min at each file exchange. For the final irrigation of the root canals after the shaping process, 5 ml of 5.25% NaOCl, 17% EDTA (Imicryl, Konya, Turkey), 5.25% NaOCl solution and isotonic saline were used in the respective order.

#### Group 2 (2.5% NaOCl)

Irrigation with 2 ml of 2.5% NaOCl solution was performed for 1 min at each file exchange. For the final irrigation of the root canals after the shaping process, 5 ml of 2.5% NaOCl, 17% EDTA (Imicryl, Konya, Turkey), 2.5% NaOCl solution and isotonic saline were used in the respective order.

#### Group 3 (1% NaOCl)

Irrigation with 2 ml of 1% NaOCl solution was performed for 1 min at each file exchange. For the final irrigation of the root canals after the shaping process, 5 ml of 1% NaOCl, 17% EDTA (Imicryl, Konya, Turkey), 5.25% NaOCl solution and isotonic saline were used in the respective order.

All irrigation procedure were performed using a NaviTip irrigation needle (30-G; Ultradent Products Inc, South Jordan, UT), placed 2 mm short of the WL. After the final irrigation process, the root canals were dried with sterile paper points that matched the canal’s diameter. All root canals were then obtured with sterile gutta-perchas (25.06 and 30.06) and AH+ (Dentsply De Trey, Konstanz, Germany) root canal sealer using the single cone technique. After root canal obturations were completed, teeth were restored with composite in a manner that ensured coronal sealing. One week after the treatment, patients were invited for a follow-up appointment. GCF samples were taken from the treated tooth in the same manner as at the beginning of the study.

### Measurement of IL-8 in GCF

Levels of IL-8 (range 5–1000 ng/L) were analyzed using a commercial enzyme-linked immunosorbent assay (ELISA) kit (IL-8 ELISA Kit [catalog no: E0089Hu]). All assay procedures were carried out according to the manufacturer’s instructions. The intra- and inter-assay coefficients of variation for IL-8 were lower than 8% and 10%, respectively. The sensitivity of the IL-8 assay was 2.51 ng/L. The results were expressed in ng/L for IL-8. To calculate the concentrations of IL-8, a standard graph was created using the curve expert 1.4 program. The IL-8 absorbance in the samples was then calculated using this standard plot.

### Statistical analysis

The data were analyzed using IBM SPSS V23. Conformity to normal distribution was examined using the Shapiro-Wilk test. The Chi-square test was used to compare categorical variables between the study groups. The Spearman’s Rho correlation coefficient was used to examine the correlation between pretreatment IL-8 levels and pretreatment pain scores. The non-parametric Mann-Whitney U test was used to compare the IL-8 levels of the inflamed tooth and the contralateral tooth. The Wilcoxon test was used to compare pre- and posttreatment IL-8 levels because the data did not follow a normal distrubition. The effect of the NaOCl solution of different concentrations on the change in IL-8 level was examined using the one-way analysis of variance (ANOVA) test.

## Results

**Fig. 1:**
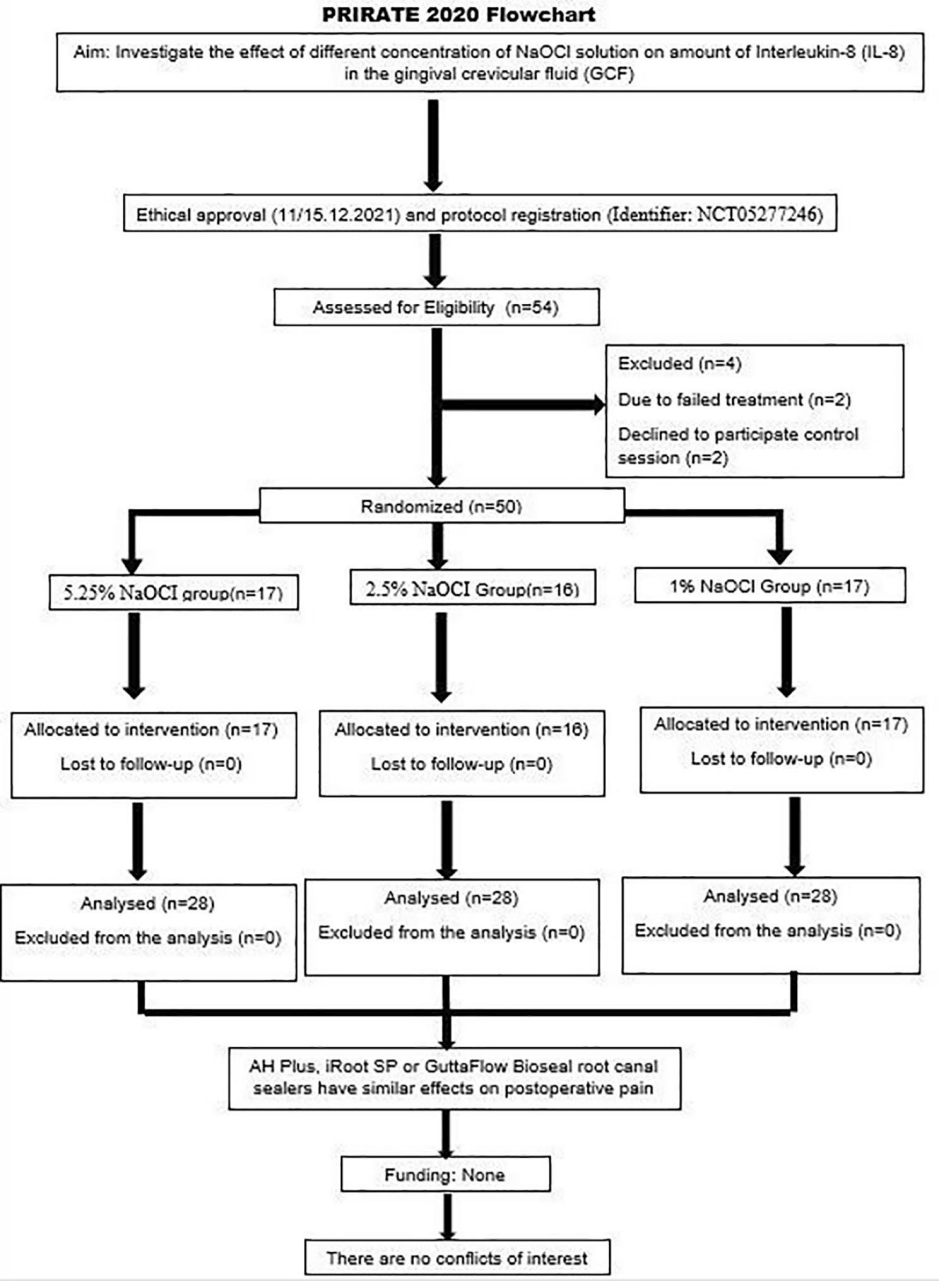
PRIRATE 2020 flowchart showing flow of participants along the study

Fifty-four patients participated in the study. However, two patients from the 2.5% NaOCl group, in whom the files were broken during root canal shaping, and two patients who did not attend the control appointment (one patient from the 5.25% NaOCI group and one patient from the 1% NaOCI group) were excluded from the final analysis (Fig. 1).

The demographic characteristics of the patients was presented on Table 1. No significant differences were found between the sex (*p* = .467), age (*p* = .774), and pretreatment pain scores (p=. 818) of the study groups. There was no correlation between pretreatment IL-8 levels and pretreatment pain scores (*p* = .521).

**Table 1:** Demographic and clinical features of the patients

	5.25% NaOCl	2.5% NaOCl	1% NaOCl	Total	p
Gender n (%)					
Female	13 (76.4)	9 (56.25)	11 (64.7)	33 (66)	0.467
Male	4 (23.6)	7 (43.75)	6 (35.3)	17 (34)	
Age (mean ± sd)	36.59 ± 8.92	33.50 ± 10.24	35.24 ± 10.17	35.14 ± 9.67	0.774
Preoperatif pain (mean ± sd)	3.59 ± 1.37	4.06 ± 1.8	3.76 ± 2.04	3.8 ± 1.73	0.818

Chi-square test statistic, frequency (percentage), mean ± standart deviation

There was a significant correlation between the IL-8 levels in the pretreatment GCF sample from the teeth with pulpitis (569.51 ± 76.94) and the IL-8 levels in the contralateral healthy teeth (444.66 ± 173.17) (*p* = .000). Furthermore, the pretreatment IL-8 levels were significantly higher than the posttreatment IL-8 levels (z=-6.154, *p* < .05).

The effect of NaOCl concentration on the change in IL-8 levels (pretreatment-posttreament IL-8) were not statistically significant (*p* > .05). The mean level of the 5.25% NaOCl group was 144.10, the 2.5% NaOCl group was 127.67, and the 1% NaOCl group was 105.30 (Table 2).

**Table 2:** Mean ± standard deviation, maximum and minimum values of IL-8 change amount

Group	mean ± sd	max	min	p
5.25% NaOCl	144.10 ± 92.52	343.31	19.64	0.376
2.5% NaOCl	127 ± 68.18	230.98	4.8	
1% NaOCl	105.302 ± 77.74	265.71	0.43	
Total	125 ± 80.34	343.31	0.43	

One Way Anova test, mean ± standart deviation, maximum and minimum

## Discussion

The antimicrobial effect of NaOCl plays a critical role in the sterilization of the root canal system and the elimination of microorganisms. As a result of this antimicrobial activity, a significant reduction in the number of microorganisms originating from the root canal system is observed. This reduction may play a role in modulating the inflammatory response and altering cytokine levels. In this context, it is anticipated that during root canal irrigation, the use of NaOCl will lead to a decrease in the levels of proinflammatory cytokines in the GCF. The primary aim of this study is to investigate this correlation. Therefore, this study investigated the effect of using different concentrations of NaOCl during root canal treatment of mandibular molars diagnosed with symptomatic irreversible pulpitis on the change in IL-8 levels in GCF. According to the study data, the effect of 5%, 2.5%, and 1% NaOCl solutions on the change of IL-8 levels was not statistically significant, and the null hypothesis of the study was accepted.

IL-8 is a member of the chemokine CXC subfamily that has a strong chemotactic effect on neutrophils and T lymphocytes. It causes the release of tissue-destructive enzymes by neutrophil degranulation. Furthermore, it affects other leukocyte types such as T cells, B cells, IL-2 activated natural killer cells, and basophils. Studies in the literature have reported that IL-8 expression is related to infection and inflammation. Karapanou et al. [8] and Park et al. [14] reported that IL-8 level in GCF markedly increased in teeth with symptomatic pulpitis compared with healthy ones. Dinçer et al. [3] also reported that the IL-8 levels in both pulp tissue and GCF of teeth with symptomatic irreversible pulpitis were significantly higher than those of healthy teeth. This study also found a significant correlation between the IL-8 level in the pretreatment GCF sample of teeth with pulpitis and the IL-8 level of the contralateral healthy teeth (*p* = .000).

There are many studies in the literature evaluating the antibacterial and antifungal activities of different concentrations of the NaOCl solution, their tissue solving capacity, debris and smear layer removal efficiency, effect on the dentin structure, dentin binding capacity, homeostatic properties, effect on endodontic instruments, effects on postoperative pain, and toxicity [11, 15, 16]. When those studies are analyzed, the common view is that NaOCl’s toxicity increases as its concentration increases, necessitating its use at the lowest effective concentration. However, despite many studies on this subject, there is still no consensus on the recommended optimal NaOCl concentration. Siqueira et al. [16] reported that all concentrations were effective, and the effect increased as concentration increased. Ayhan et al. [17] compared the antimicrobial activity of 0.5% and 5.25% NaOCl on different microorganisms and reported that the concentration of 0.5% had a significantly weaker effect. Baumgartner and Cuenin [18] reported that NaOCl concentrations of 0.5%, %1, 2.5%, and 5.25% were able to remove predentin and pulp tissue remnants from a yet unshaped canal wall. In contrast, Goldsmith et al. [19] reported that the dilution of NaOCl to 2.2% caused no meaningful reduction in tissue solvent activity; however, they reported that 0.5% NaOCl solution had an insufficient effect. We have not come across any study that addressed the effect of different concentrations of NaOCl solution on the change in IL-8 levels in GCF; thus, we had no chance to compare the results of the present study. Although our results showed that 5%, 2.5%, and 1% NaOCl solutions had no statistically significant effect on the change in IL-8 levels, the pretreatment IL-8 levels were found to be significantly higher than the posttreatment IL-8 levels. Considering these results, we believe that using NaOCl at a lower concentration would be more advantageous in terms of toxicity. It should also be noted that a decrease in the IL-8 level in the GCF occurred after the treatment, but it was not eliminated completely. Laxmi et al. [20] examined the effect of endodontic treatment on the change in IL-8 and IL-10 levels and reported that the posttreatment levels were significantly lower than the pretreatment levels, although IL-8 and IL-10 still existed. They attributed this finding to the fact that, in comprasion to other cytokines, IL-8 retains its levels at the inflammatory sites for days or even weeks.

In symptomatic irreversible pulpitis, pain occurs as intrapulpal pressure increases. The most important cause of pain is the activation of the nosiceptors surrounding the tooth [8]. Dinçer et al. reported that the levels of SP, IL-8, and MMP-8 in the pulp tissue samples were higher in teeth with acute pulpitis having a high pain score than teeth having a lower pain score [3]. Evangelin et al. [21] reported that pain intensity was significantly higher in teeth with a higher preoperative IL-8 level compared with other teeth. Also, Karapanou et al. [8] found that the GCF IL-8 level of the tooth with an intense pain score was significantly higher than that of the contralateral tooth. There are only three studies that compared pain levels and increased IL-8 expression. However, comparisons cannot be made due to the difference in the type of teeth included and the pain score examined. Therefore, we believe that further studies are needed in which the types of teeth included are standardized and more teeth with severe pain intensity are enrolled.

In endodontics, various samples such as gingival crevicular fluid, blood from the pulp, fluid derived from dentin, and periapical fluid are utilized for the analysis of inflammatory mediators [3]. Gingival crevicular fluid, synthesized by the periodontal ligament, encompasses numerous host factors including bacterial antigens, antibodies, proteins, and cytokines. The vascular and neural sources of the periodontium and endodontium are interconnected, suggesting the potential utilization of gingival crevicular fluid in evaluating endodontically-originating periapical inflammation. Particularly, the non-invasive nature of obtaining gingival crevicular fluid samples enhances the attractiveness of this diagnostic method [1, 6, 7, 9]. Studies indicate higher levels of inflammatory mediators in gingival crevicular fluid of periodontally affected teeth compared to healthy ones. Moreover, the detection of inflammatory processes in the periodontal area through gingival crevicular fluid samples has been reported [4, 5, 8]. Therefore, we believe that measuring inflammatory mediator levels in gingival crevicular fluid can be utilized in evaluating the effectiveness of treatment and patient response, thus serving as an important method for monitoring and optimizing the endodontic treatment process.

## Conclusion

Within the limits of this study, using 5.25%, 2.5%, and 1% NaOCl solutions during RCT resulted in a significant decrease in IL-8 levels. However, there was no significant difference observed between the different concentrations. We believe that it would be more advantageous to use a lower concentration of NaOCI due to the increasing toxicity associated with higher concentrations. It should also be noted that despite numerous studies on the effectiveness of NaOCl over the years, there is still no consensus on its optimum concentration. While most studies have been conducted in vitro, many in vivo studies have been subjective and focused on postoperative pain. We believe that further in vivo studies are necessary to objectively evaluate the effectiveness of NaOCI at different concentrations and reach a consensus on its optimal concentration.

## Data Availability

The datasets used and/or analysed during the current study available from the corresponding author on reasonable request.
